# Structure–Function Relationship in Citrus-Fiber-Based Emulgels for Controlled Curcumin Delivery

**DOI:** 10.3390/gels12050444

**Published:** 2026-05-19

**Authors:** Domenico Mammolenti, Domenico Gabriele, Francesca Romana Lupi, Noemi Baldino, Patrizia Formoso

**Affiliations:** 1 Department of Information, Modeling, Electronics and Systems (D.I.M.E.S.), University of Calabria, Via P. Bucci, Cubo 39C, 87036 Rende, Italy; francesca.lupi@unical.it (F.R.L.); noemi.baldino@unical.it (N.B.); 2 Department of Pharmacy, Health and Nutritional Sciences, University of Calabria, Polyfunctional Building, Via A. Savino, 87036 Rende, Italy; patrizia.formoso@unical.it

**Keywords:** insoluble dietary fiber, gelled emulsion, food viscosity, food viscoelasticity, drug delivery, polyphenols

## Abstract

Biphasic systems able to effectively release bioactive molecules along the gastrointestinal tract (GIT) are receiving growing interest. In this work, emulgels structured with citrus fiber, a digestion-resistant structuring agent, were produced using two types of edible oils (Miglyol^®^ 812 N and rice oil). Samples with 3% *w*/*w* of fiber were loaded with curcumin. The rheology of emulgels, reference hydrogels, and oil phases was studied. Complex modulus ($G^{*}$) and viscosity (η) increased with increasing fiber fraction, whereas the phase angle (δ) was fiber fraction-independent (*p* < 0.05). Dynamic and flow behaviors were modeled using weak gel model and modified Cross model, respectively. Samples with rice oil were more consistent and viscous than samples with Miglyol^®^ 812 N because of the higher $G^{*}$ and η of rice oil. Curcumin does not affect the rheology of oils, whereas it modifies the emulgel behavior. In emulgels, curcumin does not change (*p* < 0.005) both weak gel parameters. Gel strength (A) was 750 ± 40 Pa s^z^ again 760 ± 40 Pa s^z^ and 597 ± 2 Pa s^z^ again 604 ± 4 Pa s^z^ for the system with rice oil and Miglyol^®^ 812 N, respectively, and network extension (n) resulted to be 14.13 ± 0.03 for all samples. Curcumin slightly increases the phase angle δ, 5.83 ± 0.09° again 7.0 ± 0.2° and 5.5 ± 0.1° again 7.10 ± 0.08° for the system with rice oil and Miglyol^®^ 812 N, respectively. This suggests a reduction in the structure of the fiber network. Curcumin has an oil-dependent influence on the zero-shear-rate viscosity (${µ}_{0}$) and on the time constant (m), while it does not affect the shear-thinning index (n), which resulted to be statistically independent of all systems (*p* < 0.05) yielding an average value of 1.616 ± 0.007. According to in vitro release studies, the percentage of cumulative released curcumin at 24 h was 15 ± 1% for emulgel with Miglyol^®^ 812 N, whereas for the sample with rice oil, it was 18 ± 1%. Overall, results suggest the attractiveness of these systems for potential applications in the sustained oral release of curcumin.

## 1. Introduction

Owing to their characteristics, such as the possibility to encapsulate both hydrophilic and/or hydrophobic molecules, food-grade emulgels are considered a good oral delivery system for the release of active molecules [1,2,3]. Generally speaking, emulgels are O/W systems in which oil droplets are surrounded by a water phase structured by a network of polymer chains or particles [1,2]. Concerning the emulgels, as delivery systems for food applications, there is a strong interest in both delivering new active compounds [3,4] and on the development of the carrier systems itself. For the latter, the research on gelling agents and on the properties that they provide to the whole systems, such as rheological features, morphology, and physical stability, represent key factors [3,4]. In particular, on one hand they are relevant for a comprehensive interpretation of release kinetics and, on the other, they are necessary for a systematic process scale-up. It is worth saying that, recently, in the research on emulgel-type carrier systems, good results were obtained even in the utilization of combined biopolymers (complexed), like in the formation of Pickering emulgels stabilized with phycocyanin-cation starch nanoparticles [5] or with chitin nanofibril-cellulose nanocrystals [6]. However, because of their intrinsic resistance to digestion, different works consider simple biopolymers like polysaccharides or natural polysaccharide mixtures for the development of systems capable of delivering bioactives in GIT [1,2,3,4,7]. For instance, delivery systems based on dietary fibers are recognized as particularly suitable for polyphenol delivery in the colon [7].

In the present work, emulgel formulations suitable for potential application as oral carriers for the delivery of curcumin in the GIT are developed. Insoluble dietary fiber from citrus fruits was used as a structing agent and curcumin was used as active compound, whereas two oils with different characteristics, i.e., a commercial rice oil and Miglyol^®^ 812 N, were used, aiming at investigating the potential influence of the oil on emulgel properties and curcumin release. In particular, rice oil is a mixture of long-chain triglycerides (LCTs) such as oleic, linoleic, and palmitic acids [8], also containing a relevant amount of vitamin E [8]. On the contrary, Miglyol^®^ 812 N is an odorless and tasteless mixture of medium-chain triglycerides (MCTs), specifically glycerol triester of caprylic and capric acid [9,10]. Citrus fiber is an insoluble dietary fiber, obtained by processing citrus by-products, having interesting physiological benefits (e.g., improving gastrointestinal transit, reduction in risk of developing gastrointestinal disorders) linked to its resistance to digestion and competitive technological properties (e.g., ability of structuring aqueous phase) mainly linked to the physicochemical profile of fiber and processing [11,12]. Nowadays, it is considered a particularly attractive food ingredient, suitable for the preparation of different types of biphasic systems for food applications [12], such as emulgel [13] and bigels [14] or Pickering emulsions. In addition to its physiological functions and technological characteristics, citrus fiber offers even more benefits. Since it is obtained from the valorization of peels coming from citrus processing as a by-product, in contrast to some gums or other polysaccharides, citrus fiber represents a real sustainable and circular food ingredient [12]. In addition, from a commercial point of view, insoluble dietary fiber, like citrus fiber, is more attractive when comparing it to other food gelling agents, since they are not codified as additives (with an E-number), but rather as (simple) ingredients, promoting the formulation of clean labeled foodstuff [10]. Finally, curcumin is a lipophilic polyphenolic molecule obtained from the turmeric (*Curcuma longa*) rhizome, responsible for different health benefits [10,15], such as anti-oxidant, ant-inflammatory, anti-microbial, and anti-cancer properties [10,15]. Because of its hydrophobicity and poor stability in water and water-based medium, curcumin needs a non-polar medium to be dissolved [10,15,16]. Despite the dissolution in non-polar solvents such as edible oils, curcumin remains rather difficult to administer orally in an effective way, since it is instable within the GIT and undergoes rapid elimination through metabolism [15]. From here, there is a need to properly encapsulate it inside the oil droplet phase of biphasic formulations able to effectively deliver it orally [15,16]. It is worth noting that the entrapment of curcumin in different types of biphasic delivery systems like Pickering emulsion [17], nanoemulsion [10,18], oleogel [19], oleogel-based nanoemulsion [20], emulgel [16], emulgel particles [15], and gelled emulsion filled with solid lipid particles (bigels) [21] is recently receiving a lot of attention. In particular, concerning biphasic systems, it was observed that the encapsulation efficiency and curcumin bio-accessibility of Pickering emulsions stabilized by different types of rice glutelin fibril fractions notably depends on the particle type [17]. Curcumin was successfully encapsulated in nanoemulsions of Miglyol^®^ 812 N stabilized by β-lactoglobulin, suitable for topical release [10]. From the study, it turns out that curcumin does not modify the droplet size and physical stability of the whole systems. The effects of different types of surfactants (Tween 20, soy lecithin, and sucrose monopalmitate) in the formulation of curcumin-loaded nanoemulsions was also investigated [18], reporting that besides affecting physicochemical properties and stability of the systems, the surfactant type notably affects the encapsulation efficiency, loading capacity, and antioxidant activity of the curcumin-loaded nanoemulsions. The utilization of monoglyceride in structuring the oil droplets of nanoemulsions loaded with curcumin resulted a potential way to increase encapsulation efficiency and loading capacity of the systems and, at the same time, to slow curcumin delivery [20]. Emulgels structured with rhamnogalacturonan-I-enriched pectin were recognized as very effective carriers of curcumin [16], by providing higher protection of the curcumin loaded in soybean oil droplets, slowing its release but without affecting its bioaccessibility.

The present work aims at the development of emulgel formulations structured with citrus fiber and suitable for the potential oral release of curcumin. In particular, we would like to use the intrinsic resistance to digestion of citrus-fiber-particle gel matrix to potentially allow sustained release of oil droplet-entrapped curcumin in the whole GIT, even up to the colon. The usage of cellulose-based material in emulsion-type carriers for the delivery of an anti-inflammatory in the colon was also recently explored in the case of Pickering emulgel stabilized by cellulose nanocrystals and gelled via α-cyclodextrin/polyethylene glycol network [22]. In particular, such a type of carrier is promising in the localized colonic delivery of budesonide [22]. Aiming at having a deeper understanding of emulgel behavior, the physical and functional properties of citrus fiber were also investigated before emulgel production. The development of emulgels was carried out with a multiscale approach and by funding the study on rheological investigations and on further physicochemical characterizations aimed at assessing physical stability and morphology. In particular, frequency sweep and flow curve tests were used to evaluate dynamic and flow behavior of samples, respectively. Zeta potential measurements over time and at three distinct temperatures (25, 37, and 50 °C) were carried out for stability evaluation, whereas contrast phase optical microscopy and cryo-SEM microscopy were used for the morphology investigations. The microstructural differences among samples were described using the droplet size distribution (DSD) that was obtained from the analysis of the images of diluted samples. To better understand the properties of samples, single phases, i.e., fiber-based particle gel, oils, and curcumin-loaded oils, were also investigated. Specifically, frequency sweep and flow curve tests were performed on oils, curcumin-loaded oils, and fiber gel, and for the latter, the morphology was also studied via contrast phase microscopy and cryo-SEM. In addition, entrapment efficiency and loading capacity were estimated, and the ability of the emulgel system to release curcumin was explored, although in a preliminary form, by carrying out in vitro release experiments with a Franz cell for 24 h and by modeling the results using different kinetic models.

## 2. Results and Discussion

### 2.1. Physical and Functional Properties and Morphology of Citrus Fiber

The values of humidity, bulk density, particle size, zeta potential (ζ), water holding capacity (WHC), water swelling capacity (WSC), and oil-holding capacity (OHC) of the sample (citrus fiber powder) are reported in Table 1. The physical (humidity, bulk density, particle size) and functional (WHC, WSC) proprieties of the citrus fiber sample were very close to those already found in the literature for similar systems [23,24]. In particular, WHC and WSC found for a similar citrus fiber were 16.1 ± 0.6 g/g and 16.1 ± 0.4 mL/g [24], while those of modified/unmodified soybean fiber were 10.33 ± 0.15–39.64 ± 0.03 g/g and 4.58 ± 0.08–41.40 ± 0.16 mL/g [23]. In general, the values found in the present study are within the typical range reported in the literature (WHC = 6.4–74.3 g/g, WSC = 0.11–62.2 mL/g) [12,25]. The OHC value of sample towards rice oil and Miglyol^®^ 812 N is almost independent of the lipid matrix (see Table 1) and approximately equal to 2.9 g/g. These values of OHC fall within the typical range reported in the literature for insoluble dietary fiber (1–56 g/g) [25]. Finally, Looking at Table 1, it is possible to conclude that the addition of curcumin to the oil phase does not yield any change in OHC.

Concerning ζ, a value of −31 ± 1 mV (determined in a suspension with pH = 5.8 ± 0.1, conductivity = 122.5 ± 0.3 μS/cm, pH/Ion meter SevenDirect SD50, Mettler Toledo, Columbus, OH, USA) was found, indicating a strongly negatively charged surface and a consequent stable electrostatic interfacial layer [24,26,27]. The value found is in line with those typically found in literature, as can be seen considering some representative ζ data of different types of insoluble dietary fibers reported in Table S1 of the Supplementary Materials. Looking at Table S1, it is worth mentioning that, for insoluble dietary fibers, ζ is strictly affected by modifications (chemical and physical) and the medium composition (presence of salts for instance).

**Figure 1:**
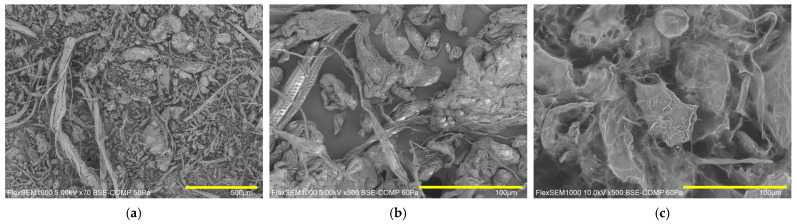
Morphology of citrus fiber powder: (**a**) SEM micrograph of citrus fiber powder at a magnification of 70×, (**b**) SEM micrograph of citrus fiber powder at a magnification of 500×, and (**c**) Cryo-SEM micrograph of hydrogel containing 3% *w*/*w* of citrus fiber (H3) at a magnification of 500×. For figure (**a**), the bar corresponds to 500 µm, whereas for (**b**,**c**), it corresponds to 100 µm.

Citrus fibers exhibited a wide and diversified range of size and shape (Figure 1a) spacings from long fibrils (several hundreds of microns) to small globular fiber (few microns), passing from short fibrils and large globular fiber particles, in agreement with the information reported in the literature for a similar fiber sample [28]. Qualitatively, the size of fiber, appreciable in Figure 1a, agrees with the data reported in Table 1. From Figure 1b, it is easier to observe how both fibrillar and globular particles have a very rough surface in agreement with the literature [24,28], indicating a high surface area and a good tendency of water sorption, hydration, and swelling. Comparing Figure 1b,c, it is possible to see that the homogenization process changes the fiber morphology by opening their structure and inducing swelling, in agreement with the literature [24,28].

### 2.2. Dynamic Oscillatory Behavior of Single Phases and Unloaded Emulgels

The results of frequency sweep tests on particle hydrogels at different fiber concentrations (2% *w*/*w*, 2.5% *w*/*w*, and 3% *w*/*w*) named H2, H2.5, and H3 are reported in Figure S1a of the Supplementary Materials (SMs). Citrus fiber structured gel showed typical weak gel behavior (δ~10° for all samples) [29] and $G^{*}$ increases (more than linearly) with the fiber content, as already found in previous works for hydrogels structured with citrus fiber, even in the presence of a solute in the water medium [13,24].

It is worth remembering that citrus fiber is mainly made of insoluble fraction and when it is dispersed in water, under vigorous mixing, insoluble particles are able to adsorb water, swelling and interacting with each other, yielding the formation of a “particle gel” [24,30,31] having properties depending on the fiber amount and homogenization power draw [32].

On the contrary, as reported in Figure S1b (Supplementary Materials), the frequency sweep test on oil samples showed ideal viscous behavior, δ = 90°. Rice oils showed $G^{*}$ higher than that of Miglyol^®^ 812 N. This difference is connected to the difference in the chemical profile of the oils; indeed, the first is a mixture of long-chain triglycerides (LCTs) and the second a mixture of medium-chain triglycerides (MCTs). According to the literature, the longer the chain, the higher the consistency [33,34], and consequently, a mixture of LCTs (rice oil) shows $G^{*}$ higher than that of a mixture of MCTs (Miglyol^®^ 812 N).

**Figure 2:**
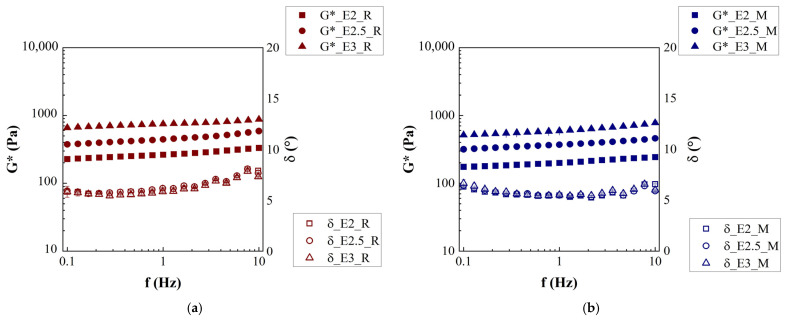
Frequency sweep test of emulgel: (**a**) samples with rice oil and (**b**) samples with Miglyol^®^ 812 N.

The data obtained from the frequency sweep test of all emulgel samples, in terms of $G^{*}$ and δ, as a function of frequency, are reported in Figure 2a,b. All samples (with both oils) exhibit solid-like behavior since δ < 45°. $G^{*}$ increases with increasing fiber fraction in the water phase, as already observed in the previous works on similar emulgel [12,13].

When biphasic systems are obtained, starting from fiber particle gels, the oil droplets are entrapped within the gel network made by particle interactions, and an emulgel is obtained having properties depending on the dispersing phase (i.e., the particle gel), dispersed phase (i.e., oil nature), and their ratio.

Comparing Figure 2a,b, samples with rice oil are more consistent than that with Miglyol^®^ 812 N, in agreement with the results of the frequency sweep test carried out on oils and oil phases (Figure S1b in Supplementary Materials), which show a greater complex modulus of rice oil with respect to Miglyol^®^ 812 N. This suggests that more viscous inclusions yield more consistent biphasic systems according to the rheological models commonly proposed to describe the behavior of these systems where the ratio of the stiffness of the matrix and the stiffness of the filler particles is a parameter controlling the macroscopic behavior of the system [14,35]. Looking at the values of δ, it is possible to say that all samples show a similar structuring degree (δ~6°) and, in particular, weak gel behavior. Overall, the consistency of emulgel samples, similarly to that of the corresponding water phase, increases with the increasing fiber fraction in the water phase. This is expected according to the literature highlighting that those properties of emulgels are determined mainly by the network properties of the continuous phase [35].

Frequency sweep tests were modeled by fitting the data with weak gel model (Equation (6)), and obtained parameters, i.e., gel strength (A) and network extension (z), are reported in Table 2, together with phase angle (δ). Gel strength increases with an increasing fiber content in the emulgel, and samples with rice oil present higher values of A compared to those of samples containing Miglyol^®^ 812 N, confirming the previous considerations on the effects of fiber on particle gel behavior and in turn the effects of the continuous phase on emulgel characteristics.

There is no statistically significative difference among the z values, of tested emulgels, that range between 13.53 ± 0.04 and 18 ± 2. The notable increase in gel strength with fiber concentration is rather expected since it was previously observed in $G^{*}$ at 1 Hz for biphasic systems containing a similar fiber, i.e., citrus fiber-based emulgel, produced with the same device and similar conditions [13]. Concerning δ, the values reported in Table 2 indicate that it mainly depends on the oil type; samples with rice oil are slightly less structured, while it does not depend on fiber fraction.

### 2.3. Flow Behavior of Single Phases and Unloaded Emulgel

**Figure 3:**
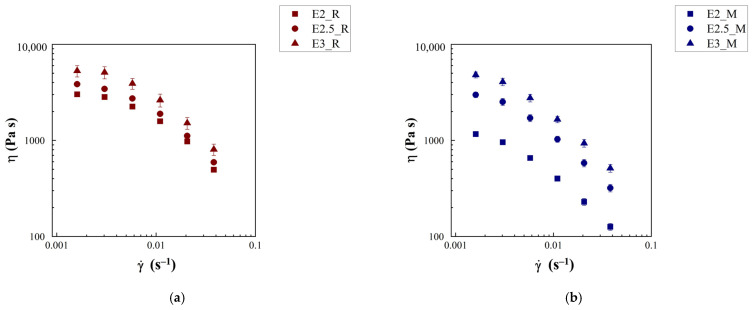
Flow curve of emulgel: (**a**) samples with rice oil and (**b**) samples with Miglyol ^®^ 812 N.

Flow curve tests (Figure 3a,b) evidenced, for all emulgels, a shear thinning behavior with a zero-shear viscosity plateau, more or less evident depending on the fiber content. The shear thinning behavior is typical for food emulsions like emulgels, nanoemulsions, or Pickering emulsions, as reported in other works [9,10]. Emulgel with 3% *w*/*w* of fiber (in water phase) was found to be more viscous than the others and showed a more visible onset of the zero-shear viscosity plateau (emulgels with rice oil, Figure 3a, and emulgels with Miglyol^®^ 812 N, Figure 3b). It is worth noticing that for shear rate values higher than 0.04 s^−1^, instability phenomena (e. g., edge fracture) occurred for all the samples making the measured values not reliable. Shear thinning behavior, zero-shear viscosity plateau, and edge fracture at high shear rates were also found for hydrogel samples, as reported in Figure S2c, confirming that the flow properties of emulgels are also mainly due to the fiber network. Comparing emulgel samples (Figure 3) to particle hydrogel samples (Figure S2c), the latter show more pronounced differences related to fiber fraction. For higher-concentration systems (H2.5 and H3), instability appears at lower shear rates. It is quite evident that samples with rice oil (Figure 3a) are more viscous than those with Miglyol^®^ 812 N (Figure 3b), in agreement with results observed for the oil phases reported in Figure S1d. Indeed, rice oil is more viscous than Miglyol^®^ 812 N, 75 ± 1 mPa s and 25 ± 1 mPa s, respectively. It is worth noting that the value of viscosity found for Miglyol^®^ 812 N (calculated as the average of the values reported in Figure S1d) is in agreement with that provided by the manufacturer (25–33 mPa at 20 °C), and that of rice oil (calculated as the average of the values reported in Figure S1d) is in line with the literature data [34,36]. The difference in viscosity between rice oil and Miglyol^®^ 812 N, due to the very different chemical profile, is rather expected since, according to literature, the longer the chain, the higher the viscosity [33,34]; consequently, a mixture of LCTs (rice oil) shows a higher η than a mixture of MCTs (Miglyol^®^ 812 N).

The parameters obtained by fitting viscosity data with modified Cross model (Equation (5)), i.e., zero-shear-rate viscosity (${µ}_{0}$), time constant (m), and shear-thinning index (n), are reported in Table 3. For both sets of samples, increasing the fiber fraction ${µ}_{0}$ increases, whereas m is independent of the fiber amount, but it is dependent on oil nature, being lower for samples containing rice oil. Looking at Table 3, ${µ}_{0}$ of samples with rice oil is tendentially higher than that of samples with Miglyol^®^ 812 N. Finally, the values of n reported in Table 3 do not suggest any correlation with the fiber content or oil type since there are no significant differences (*p* < 0.05) among them from a statistical point of view.

### 2.4. Analysis of Zeta Potential in Emulgel Samples

The physical stability of emulgels was investigated within a period of 4 weeks and at different temperatures, measuring the zeta potential (ζ). The zeta potential is the electrical potential at the slipping plane of the interfacial double layer surrounding the o/w droplets [26]. According to the literature, large absolute values of zeta potential (≥30 mV), being related to relevant repulsion forces, suggest good stability of the biphasic systems [26].

**Figure 4:**
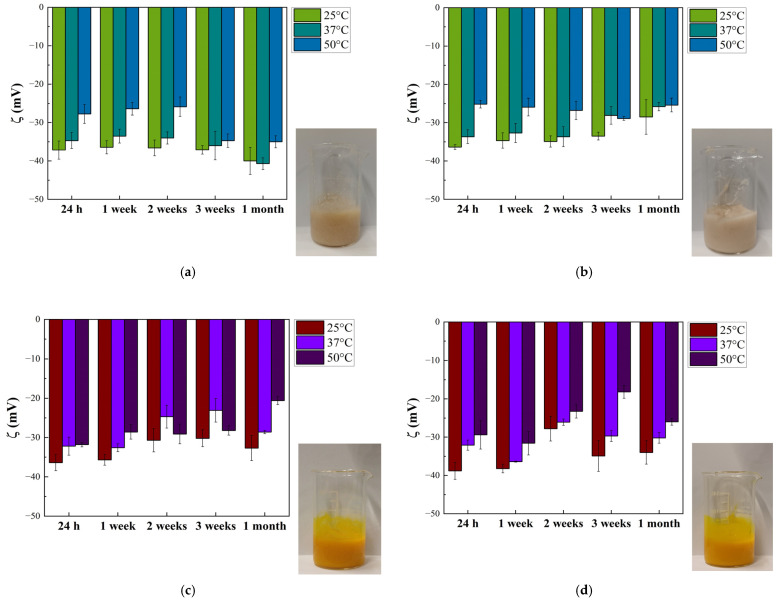
Analysis of ζ in emulgels samples: (**a**) ζ of E3_R over time and at different temperatures, (**b**) ζ of E3_M over time and at different temperatures, (**c**), ζ of E3_R/C over time and at different temperatures, and (**d**) ζ of E3_M/C over time and at different temperatures.

Zeta potential absolute values for unloaded emulgels E3_R and E3_M are shown in Figure 4a,b, whereas data for samples with lower fiber concentrations (E2_R, E2.5_R, and E2_M, E2.5_M) are shown in Figure S2a,b and Figure S3a,b, respectively. As Figure 4a,b suggests, the absolute zeta potential of emulgels prepared with different oils exceeded 30 mV and showed no significant (*p* < 0.05) variations over time, regardless of the temperature, except at 50 °C. A slight decrease in ζ of the E3_M emulgel was also noted at 37 °C after 3 weeks of storage. This indicates that both emulgel systems exhibited high stability against flocculation and coalescence for at least 1 month from the preparation [24,26,27].

As illustrated in Figures S2 and S3, both emulgel systems with lower concentrations of citrus fiber exhibited slightly different ζ trends. However, in general, the ζ of sample with rice oil (Figure S2) and Miglyol^®^ 812 N (Figure S3) remained consistently around −30 mV, suggesting good stability [26]. It is interesting to observe that the ζ values found for all the emulgels are also comparable to that of citrus fiber (Table 2) and to values reported in the literature for citrus fiber particle gels [24]. Then, the fiber fraction of 0.03 *w*/*w* was chosen for the preparation of curcumin-loaded emulgel samples because its rheological properties, mainly $G^{*}$, are close to those of commercial emulsion-based foods [13,37,38,39]. Based on these considerations, emulgel samples containing 3% *w*/*w* of fiber were prepared using curcumin-loaded rice oil and Miglyol^®^ 812 N, named as E3_R/C and E3_M/C, respectively. In sample E3_R/C, the curcumin concentration in the oil phase was 2.67 mg/ml, whereas in E3_M/C, it was 3.56 mg/ml. The different concentrations of curcumin in the two emulgel formulations were chosen based on the specific saturation solubility of the bioactive in each oil phase. This approach was aimed at maximizing the bioaccessibility of curcumin. The ζ values at different temperatures (25, 37, and 50 °C) over a one-month storage period (after 24 h, 1 week, 2 weeks, 3 weeks, and 1 month from preparation) are reported in Figure 4c,d. The behavior found for the analogous non-curcumin-loaded samples (E3_R and E3_M) remained unaltered by the entrapment of curcumin, as suggested by previous reports on curcumin-loaded emulsion-type systems [10,18]. Indeed, for both curcumin-loaded emulgels, E3_R/C and E3_M/C, most of the ζ values are approximately −30 mV. Minor exceptions were observed at high temperatures, consistent with the behavior of the unloaded systems, yet more pronounced for E3_R/C at 50 °C.

### 2.5. Microstructure of Emulgel Samples

**Figure 5:**
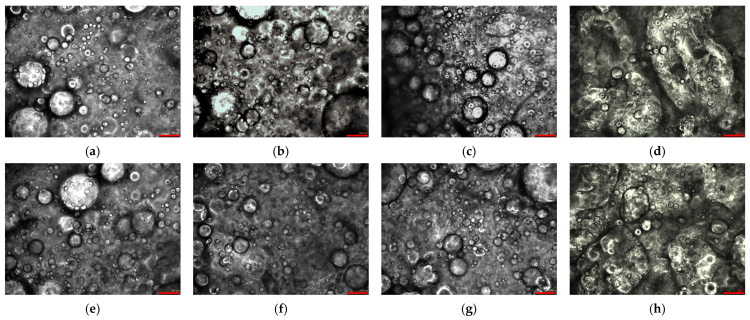
Contrast phase micrographs of unloaded emulgel samples at different fiber concentrations and oil types: (**a**) E2_R, (**b**) E2.5_R, (**c**) E3_R, (**d**) E3_R/C, (**e**) E2_M, (**f**) E2.5_M, (**g**) E3_M, and (**h**) E3_M/C. the bar corresponds to 100 µm.

As reported in the micrographs in Figure 5a–c,e–g, all the unloaded emulgels showed a uniform morphology with a uniform spatial distribution of oil droplets in the gel matrix. These results are similar to those obtained for similar emulgels [13,40]. From a qualitative viewpoint, all the micrographs evidence the presence of large droplets and a very wide droplet size distribution. In general, emulgels exhibited droplets with a size in the order of 10–100 µm. No particular change in morphology, related to either fiber concentration or oil type, can be appreciated. It is worth saying that, in the explored range of composition, the independence of morphology from the fiber fraction is rather expected, according to the results already obtained for similar emulgels produced with sunflower oil and lecithin as oil phase [13] by using the same homogenization technique in similar conditions. Comparing Figure 5c (sample E3_R) with Figure 5d (sample E3_R/C) and Figure 5g (sample E3_M) with Figure 5h (sample E3_M/C), it is possible to conclude that curcumin addition does not affect the morphology of systems, at least at this scale of observation.

The equivalent area circular diameter and droplet size distribution (DSD) were calculated by processing the micrographs taken of diluted samples. The results in terms of the 10th percentile diameters ($d_{10}$), median diameter ($d_{50}$), 90th percentile diameter ($d_{90}$),and SPAN index (SPAN), which provide a quantitative tool for microstructure analysis, are reported in Table 4. No statistical difference (*p* < 0.05) was observed for $d_{10}$, whereas, even though some differences were observed for $d_{50}$, $d_{90}$, and SPAN, no clear trend and very similar values were found. Overall, the droplet size seems to be independent of both the oil type and fiber fraction. A similar droplet size was also found by Bruno et al. 2022 [13] for an emulgel structured with different fractions of a similar fiber and produced with a similar device, although the DSD was quantified based on different parameters (mean diameter and standard deviation). It can be speculated that keeping constant the oil fraction, the adopted homogenization conditions and the corresponding power draw are suitable to allow a sort of saturation condition in the oil dispersion yielding, as a final result, only small changes in DSD that are not statistically significant.

Even samples loaded with curcumin (E3_R/C and E3_M/C) did not show significant differences with respect to all other samples (*p* < 0.05). These results agree with data obtained for a nanoemulsion loaded with curcumin and stabilized by β-lactoglobulin; in this case, the authors observed that curcumin does not affect the mean diameter of the oil phase for at least 80 days [9]. In general, for different types of curcumin-loaded biphasic systems such as a nanoemulsion and emulgel, it was observed that the diameter of the oil phase droplets is more affected by other factors, like the emulsifier type and concentration or processing conditions, rather than curcumin loading [10,18,20].

**Figure 6:**
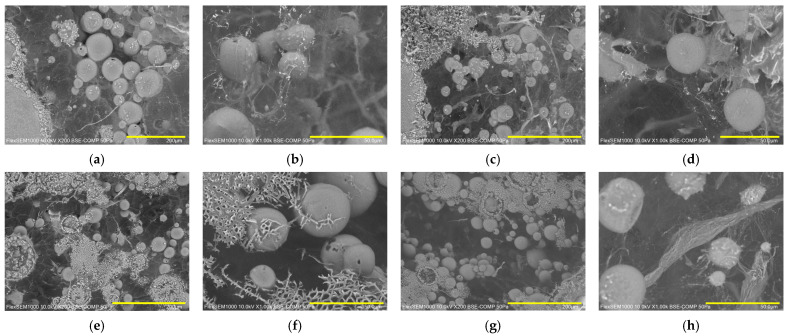
Cryo-SEM images of unloaded and loaded emulgel samples: (**a**) E3_R at 200×, (**b**) E3_R at 1000×, (**c**) E3_R/C at 200×, (**d**) E3_R/C at 1000×, (**e**) E3_M at 200×, (**f**) E3_M at 1000×, (**g**) E3_M/C at 200×, and (**h**) E3_M/C at 1000×. For images at 200×, the bar corresponds to 200 µm, whereas for images at 1000×, it corresponds to 50 µm.

Further consideration of the microstructure of samples can be based on SEM images in Figure 6a–f, which reports the morphology of diluted samples E3_R, E3_R/C, E3_M, and E3_M/C. Comparing Figure 6a (sample E3_R) and Figure 6e (sample E3_M), it is possible to see that samples with different oils showed similar microstructures, and some droplet aggregation can be seen, even after sample dilution.

The similarity between samples E3_R and E3_M can be appreciated also at a smaller scale, as reported in Figure 6b,f. The addition of curcumin seems to not affect the observed microstructure, at least in the investigated conditions, as evidenced by comparing images of loaded samples E3_R/C and E3_M/C (Figure 6c,d,g,h) to those of matrices without curcumin, E3_R and E3_M, (Figure 6a,b,e,f), at both investigated magnifications. Overall, the SEM images confirm the morphological/microstructural findings of optical microscopy and DSD analyses.

### 2.6. Effect of Curcumin Loading on Dynamic Oscillatory Behavior of Emulgel

**Figure 7:**
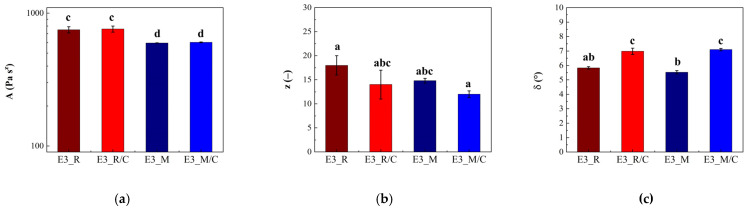
Dynamic oscillatory behavior of loaded and unloaded emulgel samples: (**a**) gel strength, (**b**) network extension, and (**c**) phase angle at 1 Hz. Different letters, for the same parameter, refer to significantly different values. The letters refer to and complete the notation shown in Table 2.

The comparison of frequency sweep tests on loaded and unloaded oils is reported in Figure S1b; from the plot, it is possible to conclude that curcumin loading does not exert any action on $G^{*}$ and δ in linear conditions in the whole range of frequencies on both rice oil and Miglyol^®^ 812 N. Gel strength (A), network extension (z), and phase angle (δ) at 1 Hz, are reported in Figure 7a–c. In Figure 7, the letters above the histogram refer to the ANOVA analysis, which was carried out for each parameter (A, z), on the whole population of data, i.e., all the emulgel samples, both loaded and unloaded.

From Figure 7a, no significant differences (*p* < 0.05) can be seen either between the value of A of sample E3_R/C and that of E3_R or between those of sample E3_M/C and E3_M, indicating that the addition of curcumin does not affect the values of gel strength. The network extension values of samples E3_R, E3_R/C, E3_M, and E3_M/C (Figure 7b) do not show significant differences even though the curcumin addition seems to yield a non-significant reduction in the z value. This trend is in agreement with phase angle results at 1 Hz (Figure 7c) where a significant increase with the addition of curcumin in the oil droplets of emulgels (comparison of E3_R with E3_R/C and E3_M with E3_M/C) is observed, confirming an increase in liquid-like behavior and, therefore, a reduction in the structuring degree. The reduction in the structuring degree is rather unexpected, since no effects on $G^{*}$ and δ were found when comparing pure oils to curcumin oil solutions, Figure S1b. Even though different aspects of curcumin-added systems (e.g., effects of surfactants, particle type or organogelator, on rheological properties of matrices [10,17,18,19,41]) were explored in the literature, few data are available about the effects of the curcumin content, and they do not suggest any direct effect. For instance, for surfactants-based mesophases with different compositions (water: 30–50%, oleic acid: 10–30%, surfactant 40%), no effect connected to curcumin addition (5 mg/g) was observed in terms of $G^{\prime }$ and $G^{\prime \prime }$ by Fonseca-Santos et al. [42]. Even mechanical properties coming from the texture analysis (hardness, compressibility, adhesiveness, cohesiveness) were found to be independent of curcumin addition.

It could be speculated that the reduction in structuring degree observed in the present work could be related to potential curcumin interactions with the gel matrix or at the O/W interface between the gel matrix and the oil-phase droplets. Indeed, curcumin can be bonded by cellulose-based materials, as reported by Zainuddin et al. [43], even if greater values of curcumin-binding capacity were found for hydrophobically modified nanocrystalline cellulose rather than for native nanocrystalline cellulose. Furthermore, curcumin exerts different types of interfacial effects as reported by different authors [10,17,18,19,39]. Specifically, on one hand, it was reported that curcumin can decrease the interfacial tension of oily systems acting as a weak surfactant [10,44]; on the other hand, as reported for the W/O system by Del Duca et al. [45], for the O/W system by Aditya et al. [46], curcumin can even form sub-micron particles (both amorphous and crystalline) via anti-solvent precipitation. Nevertheless, for an exhaustive and deeper analysis of curcumin bonding and interfacial behavior, further studies with the due detail are required to better highlight the potential mechanism behind the observed weakening effects due to curcumin addition.

### 2.7. Effect of Curcumin Loading on Flow Behavior of Emulgels

**Figure 8:**
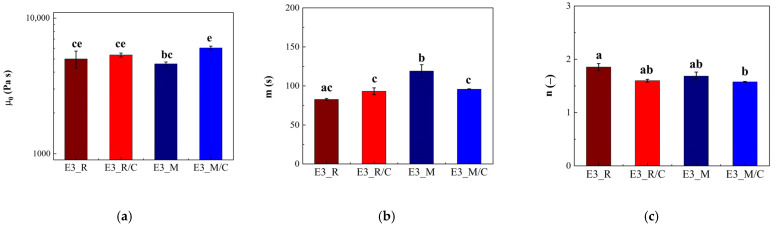
Flow behavior of loaded and unloaded emulgel samples: (**a**) zero-shear-rate viscosity, (**b**) time constant and (**c**) shear-thinning index. Different letters, for the same parameter, refer to significantly different values. The letters refer to and complete the notation shown in Table 3.

The value of the zero-shear-rate viscosity (${µ}_{0}$), time constant (m), and shear-thinning index (n) of curcumin-added samples are reported in Figure 8a–c. In Figure 8, the letters above the histograms refer to the ANOVA analysis, which was carried out for each parameter (${µ}_{0}$, m, n), on the whole population of data, i.e., all the emulgel samples, both loaded and unloaded.

Looking at Figure 8a, it is possible to see that ${µ}_{0}$ of the samples with rice oil with and without curcumin (E3_R and E3_R/C) are similar and higher than that of sample E3_M, whereas sample E3_M/C showed the highest value. Concerning m (Figure 8b), no difference was observed between rice oils samples (with and without curcumin), whereas for samples with Miglyol^®^ 812 N, curcumin addition yields a reduction. Finally, from Figure 8c, it is possible to see that n is almost independent from oil type and curcumin addition. In general, curcumin loading in the emulgel oil phase involves changes to the flow behavior only in the case of samples with Miglyol^®^ 812 N; in particular, it increases ${µ}_{0}$ and decreases m, but does not affect n. This suggests that the ability of curcumin to modify the flow properties of emulgels, probably related to curcumin accumulation at the interface (as previously discussed), could be either dependent on the nature of the oil or, more probably, related to the amount of added curcumin, slightly higher in the Miglyol^®^ 812 N sample. However, as mentioned above, a systematic and deep investigation (out of the aim of the present research) would be essential to fully understand the phenomenon.

### 2.8. Entrapment Efficiency and Loading Capacity of Loaded Emulgels

For emulgels, entrapment efficiency (EE%) and loading capacity (LC) were calculated according to the equations given in the Materials and Methods. The extraction method’s efficiency was validated by a complete breakdown of the emulgel structure using methanol and an exhaustive extraction protocol, which provides washing steps of the precipitated fibrous pellet after centrifugation. The values of EE% found are attributed to the strong lipophilicity of the drug, which favors its partitioning into the internal oily phase of the emulgel, and the viscosity of the structured aqueous phase, which prevents drug migration to the external aqueous phase during the emulsifying process. EE% and LC were found to be 98.2 ± 1.9% and 1.03 ± 0.03 mg/g for E3_R/C and 91.9 ± 1.3% and 1.23 ± 0.06 mg/g for E3_M/C, respectively. Similar values were previously reported for the entrapment of curcumin within soybean oil in Pickering emulsions, stabilized by glutelin fibrils [17]; specifically, the EE% was 94.17% and the LC was 1.32 mg/g for emulsions stabilized through the retained fraction of rice glutenin fibrils. Similarly, oleogel-based nanoemulsions (containing olive oil structured with monoglycerides) also showed a comparable EE% (90 ± 3% and 91 ± 3% at larger amounts of organogelator); however, the LC of these systems was higher, ranging from 1.8 ± 0.0 to 6.0 ± 0.2 mg/g, due to the ability of organogelator to trap curcumin [20]. In contrast, curcumin-loaded nanoemulsions stabilized by various surfactants (Tween 20, soy lecithin, and sucrose monopalmitate) showed a lower EE%, the highest being 85.74 ± 3·10^−4^% for the sample with Tween 20, compared to the values obtained in the present study [18].

### 2.9. In Vitro Release Study

**Figure 9:**
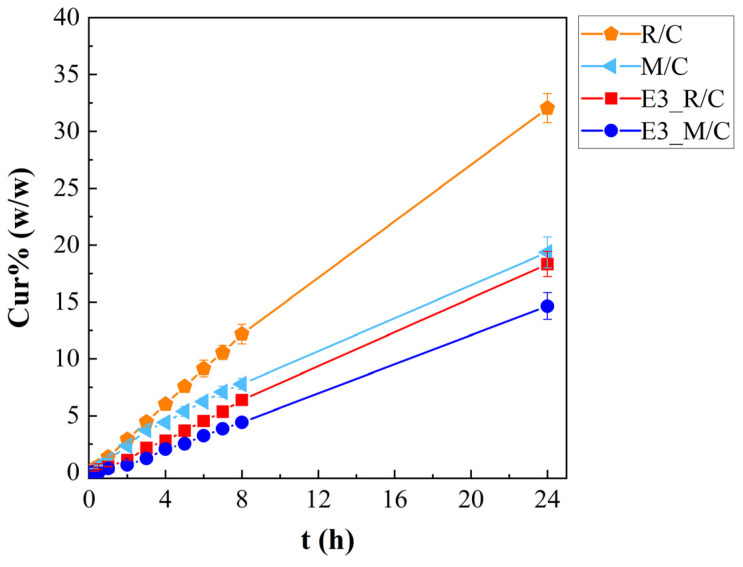
In vitro cumulative release profile of curcumin. Continuous lines are a guide for the reader.

Although dynamic digestion models simulate gastrointestinal transit, the Franz cell was chosen to specifically isolate and quantify the release profile of curcumin from the emulgel network. This step is critical for determining the fraction of the bioactive compound that becomes bio-accessible at the absorption site, allowing for a precise assessment of the emulgel efficacy as a delivery system compared to unformulated curcumin. The in vitro release analysis indicated that curcumin was gradually released from the oil phase of the citrus fiber-based emulgels, as well as from the oils alone, and was able to diffuse through a synthetic membrane, and the results are reported in Figure 9. In particular, in the figure, the Cur%, defined according to Equation (9) as the ratio between the cumulative released amount of curcumin and the total amount of curcumin in the sample multiplied by one hundred, at different times (0–24 h), is reported. The yellow coloring of curcumin was clearly observed in the receptor compartment of Franz cells after 24 h for all investigated samples.

All samples show an almost linear curcumin-release profile, with values of released curcumin slightly slower in the emulgels than in the oil solutions, since the beginning of the test. After 1 h, the release of curcumin from the R/C solution and E3_R/C emulgel showed cumulative percentages of 1.39 ± 0.07% and 0.53 ± 0.05%, reaching 12.2 ± 0.9% and 6.4 ± 0.4% after 8 h, respectively. Slightly lower values of Cur% were found for Miglyol^®^ 812 N-based samples at the same times (1.13 ± 0.05% for M/C and 0.37 ± 0.04% for E3_M/C at 1 h; 7.8 ± 0.5% and 4.4 ± 0.3% at 8 h, respectively). Furthermore, at 24 h, the cumulative release of curcumin from rice oil systems reached 32 ± 1% for R/C and 18 ± 1% for E3_R/C, respectively, whereas lower values were obtained from Miglyol^®^ 812 N analogues (19 ± 1% for M/C and 15 ± 1% for E3_M/C). The release of curcumin from fiber-based emulgels is certainly influenced by the nature of the oil phase. The formulation utilizing Miglyol^®^812 N (rich in medium-chain triglyceride, MCT) exhibited a slower and more sustained release compared to that containing rice oil (rich in long-chain triglyceride, LCT). Due to its higher solubility in Miglyol^®^ 812 N, curcumin remained more effectively partitioned within the oil droplets. Curcumin’s greater affinity for Miglyol^®^ 812 N resulted in a reservoir effect that reduced the rate of diffusion, making it preferable for sustained release. The values obtained are rather low, if compared to the release values of similar systems (evaluated with different techniques), such as curcumin loaded from the Pickering emulsion stabilized with rice glutelin fibrils [17]. It is worth noting that the higher percentage release was found in an in vitro simulated gastrointestinal digestion experiment rather than in a diffusive release experiment. It is quite evident that the sustained release of curcumin is a direct result of using citrus fiber as a structuring agent. Indeed, transmembrane flux values were 0.031 ± 0.001 mg/cm^2^ h for R/C and 0.024 ± 0.002 mg/cm^2^ h for M/C, whereas they were 0.0070 ± 0.0004 mg/cm^2^ h and 0.0057 ± 0.0005 mg/cm^2^ h for E3_R/C and E3_M/C, respectively.

In Figure S4, the cumulative released amounts of curcumin, as a function of time during the in vitro release studies, are reported. The values of transmembrane flux, obtained from Figure S4 and reported above, are rather higher if compared to that found for the nanoemulsion loaded with curcumin for topical release [10]. This is justified by the large amount of curcumin used in the emulgel in this study. However, this consideration is rather indicative, since reliable discrepancies in diffusion release parameters are possible because of differences in both the incapsulating system nature (emulgel structured with dietary fiber vs. nanoemulsion) and diffusion experimental procedures (different dissolution media composition and different dialysis membrane).

### 2.10. Release Kinetic Modeling

The release kinetics of entrapped curcumin were evaluated based on the cumulative amount of curcumin released from emulgels E3_R/C and E3_M/C and consequently diffused through the cellulose membrane into the receiving section of a vertical Franz diffusion cell. These release curves were compared to those obtained from the release study of oil solutions R/C and M/C. All in vitro release profiles of curcumin were analyzed and assessed by fitting the data of the different release kinetic models. Zero-order, first-order, Higuchi and Korsmeyer–Peppas equations [47] were used for data fitting, as graphically reported in Figure 9 and summarized in Table 5. The zero-order and first-order models are empirical kinetic models describing concentration-independent and concentration-dependent release behaviors, respectively. The Higuchi model is a theoretical model derived from Fick’s laws of diffusion. The Korsmeyer–Peppas model is a semi-empirical model used to characterize transport mechanisms involving non-Fickian diffusion [47].

Aiming at using a uniform notation, all the kinetic models were expressed in terms of $M^{\prime }(t)/M_{0}$, i.e., the ratio between cumulative amount of curcumin released at time t ($M^{\prime }(t))$ and total amount of curcumin in sample ($M_{0}$). In the Korsmeyer–Peppas model, the fraction of released curcumin (i.e., $M^{\prime }(t)$/${M^{\prime }}_{\infty }$) where ${M^{\prime }}_{\infty }$ is the amount released at infinite time) is modeled; in the present case, it was assumed that $M_{0}$ = ${M^{\prime }}_{\infty }$, i.e., the amount released at infinite time (equilibrium) is equal to the total amount in the sample; since no clear plateau was observed, this was assumed as the normalization criterion. Consequently, the Korsmeyer–Peppas model does not have its classical meaning and its model parameters became simply fitting parameters, although related to the release rate and release mechanism.

**Figure 10:**
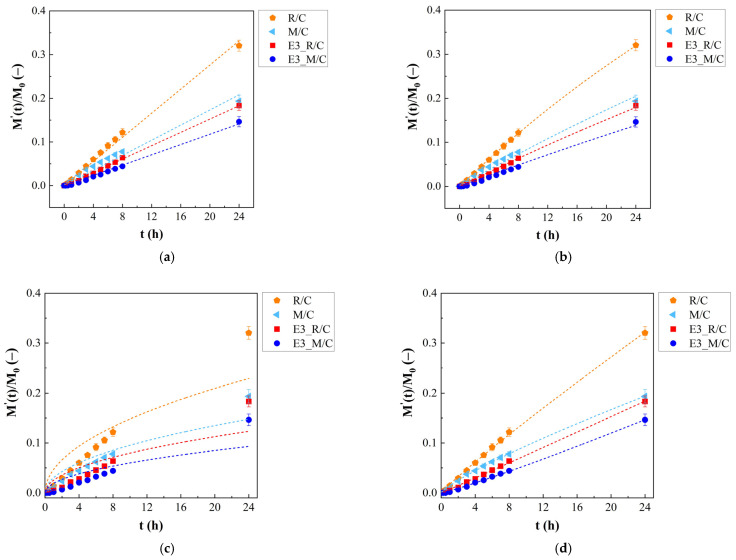
Release kinetic models: (**a**) zero-order kinetic, (**b**) first-order kinetic, (**c**) Higuchi kinetic model, and (**d**) Korsmeyer–Peppas kinetic model. Dashed lines represent the fitting curve associated with the kinetic model.

Looking at the fitting curves in Figure 9, it is possible to see that all the models fit the experimental data quite well, except for the Higuchi model (Figure 10c), which clearly shows relevant deviation for all samples. This suggests that the release of curcumin from both oils and emulgels is not a pure diffusive process. A detailed and quantitative analysis of the data fitting is reported in Table 5.

In particular, for each model, the fitting parameter/s (value ± standard error), correlation coefficient (R^2^, adjusted R-square), Residual Sum of Square (RSS), Root Mean Square Error (RMSE), Akaike Information Criterion (AIC), and Bayesian Information Criterion (BIC) are reported. In particular, (adjusted) R^2^ describes the proportion of variance explained by the fitted model while accounting for the number of model parameters [48]. RSS and RMSE quantify the discrepancy between experimental and predicted values, with lower values indicating a better overall fitting accuracy [49]. AIC and BIC are likelihood-based model selection criteria that evaluate the trade-off between goodness-of-fit and the number of model parameters [50].

With the exception of the Higuchi model, all the applied models exhibit high R^2^ values, close to unity, indicating good agreement between the model and data. The standard errors based on the fitting parameters are lower than 5% for zero-order, first-order, and Korsmeyer–Peppas models but not for the Higuchi model. Tendentially, the Korsmeyer–Peppas model provides the lowest RSS, RMSE, AIC, and BIC values, indicating this as the best model for data description.

According to the value of the n parameter of the Korsmeyer–Peppas model, information about the release mechanism can be obtained [47]; nevertheless, considering that the model was not used in its classical form, the considerations of the release mechanism are rather indicative and would require further experiments on a lager time scale to be verified. Specifically, for solutions, a value of n slightly lower than 1 was found, while for emulgels, n was slightly higher than 1. This suggests that some sort of Case II transport (involving matrix dissolution) drives the release with a tendential change towards anomalous transport in the case of R/C and M/C [47] and a tendential change towards Super Case II [47] for E3_R/C and E3_M/C where the release of the drug is probably regulated by a mixed mechanism involving diffusion and possible spatial rearrangement in citrus-fiber-based emulgels, such as fiber softening/swelling or entanglement. However, all these considerations on the release mechanism need to be assessed based on a longer experimental time where a plateau in the profile is expected. In general, solutions (R/C and M/C) showed higher releasing rate constants compared to those of emulgels (E3_R/C and E3_M/C). Furthermore, systems with rice oil (both R/C and E3_R/C) showed higher releasing rate constants compared to those of systems with Miglyol^®^ 812 N (both R/C and E3_R/C). Overall, it could be concluded that citrus-fiber-based emulgels allowed an interesting opportunity for curcumin sustained release.

## 3. Conclusions

Emulgels for curcumin sustained oral release were produced using citrus fiber as a structuring agent and rice oil and Miglyol^®^ 812 N as the oil phase. All samples exhibited satisfying stability, estimated with zeta potential values. No appreciable differences were found in the morphology and droplet size with the composition. The increase in citrus fiber increases the consistency, estimated as the complex modulus (${G\;}^{*}$) and viscosity (η) of the emulgel. On the other hand, no change was observed for the structuring degree (described by z parameter and phase angle, δ). The oil type affects consistency and viscosity but not structuring of the emulgel; particularly, samples with rice oil exhibited higher ${G\;}^{*}$ and η with respect to samples with Miglyol^®^ 812 N. The weak gel model and the modified Cross model were used to describe the rheology of emulgels, for viscoelastic and flow behavior, respectively.

Curcumin addition does not affect gel strength (described by A parameter), whereas it yields a reduction in structuring, highlighted mainly by the δ trend. Concerning the flow behavior, curcumin only affects the properties of the sample with Miglyol^®^ 812 N by increasing $\mu_{0}$ and deceasing m, suggesting that the effect of curcumin in emulgel formulations could depend either on the oil nature or on the different added amount. Results highlight that curcumin weakens the emulgel structure, even though consistency is not affected; this could be explained by a potential action of curcumin at the O/W interface. However, this hypothesis needs further experimental assessment, e.g., measurements of interfacial tension and moduli, direct observations of nanoparticles at the O/W interface, etc.

The entrapment efficiency and loading capacity of samples agree with those of similar systems already studied in the literature. A preliminary analysis of curcumin release suggests that delivery from both oil solutions and emulgel samples does not occur exclusively through diffusion. Based on kinetic model comparisons, the Korsmeyer–Peppas model provides a strong empirical fit for the data, but extended experiments are needed to gain a complete understanding of the release mechanism. Overall, according to in vitro release study, emulgel samples were particularly suitable for delayed drug delivery. The rheological, microstructural, and in vitro release results exhibited by the simple formulations developed in the present work suggest that they could be a starting point for the development of close-to commercial systems where other ingredients could be added to either modify the taste, such as salts or sweeteners, or texture and microstructure (e.g., droplet size distribution), such as emulsifiers. Prior to advancing the development of these formulations, it is essential to conduct studies that elucidate the interactions among curcumin, fiber, and the oil–water interface, as well as determine the mechanism underlying curcumin release.

## 4. Materials and Methods

### 4.1. Materials

Citrus fiber, kindly provided by JRS Silvateam Ingredients srl (Bergamo, Italy), was used as the structuring agent of the water phase. The oil phase was either rice oil (Fior di Loto srl, Villareggia, Italy) containing 40 mg/100 g of vitamins E, or Miglyol^®^ 812 N, kindly provided from Eigenmann & Veronelli SpA (Rho, Italy). Curcumin, used as the active lipophilic agent, was purchased from Sigma-Aldrich (Saint Louis, MO, USA). Methanol and ethanol were purchased from VWR International (Radnor, PA, USA). Distilled water was used as the aqueous medium.

### 4.2. Physical and Functional Properties of Dietary Fibers

The physicochemical (i.e., humidity, bulk density, water activity, particle size distribution, and color) and functional (i.e., water holding capacity (WHC), water swelling capacity (WSC), and oil holding capacity (OHC) properties of citrus fiber were investigated.

An HB43 moisture analyzer (Mettler Toledo, Columbus, OH, USA) was used to measure moisture of the fiber sample. The moisture analyzer was set at 160 °C for 5–6 min, and the final moisture value (on wet basis) was recorded when the mean weight loss per unit of time dropped below 1 mg per 50 s [24]. Bulk density was determined according to a procedure reported in the literature for the same type of material [24,51]. A graduate cylinder of 50 mL was completely filled with the citrus fiber sample and tapped against the table surface several times, until no further change in volume was observed. The volume occupied by the hydrated sample was recorded, as well as its mass. Bulk density (g/mL) was determined as a ratio of mass by volume. Water activity was measured using the AwTherm (Rotronic, Bassersdorf, Switzerland). Color was measured using a colorimeter (Croma Meter CR-400, Konica Minolta, Chiyodam, Japan), and the measurements were analyzed in the CieLab space. The color sampling was performed in triplicate using the white standard plate as background (L * = 97.02, a * = 0.14, b * = 2.26). A Mastersizer 3000 (Malvern Panalytical, Malvern, UK) equipped with the dry accessory Aero S was used to determine the particle size distribution of the citrus fiber sample.

The WHC and WSC were estimated following the procedures proposed in the literature and described in detail elsewhere [24]. Briefly, for WHC, 1 g of sample was mixed with 40 g of distilled water and left for 24 h at 25 °C. The mixture was centrifugated (5810, Eppendorf, Eppendorf, Germany) at 4000 rpm (rcf 3220× *g*) for 20 min. The supernatant was eliminated, and the (wet) mass was quantified. For the WSC, 0.5 g of sample was manually mixed with approximately 50 mL of distilled water in a graduate cylinder. The system was left to rest for 24 h at 25 °C, after which the hydrated bed volume of the fiber sample was recorded. The values of WHC and WSC were calculated according to Equations (1) and (2), respectively.

(1) $$WHC=\frac{m_{w}-m_{d}}{m_{d}}$$

(2) $$WSC=\frac{V_{h}-V_{d}}{m_{d}}$$

where $m_{w}$ and $m_{d}$ are the mass (g) of wet and dry fiber, respectively, whereas $V_{h}$ and $V_{d}$ are the volume (ml) of the hydrated and dry fiber, respectively.

For OHC, a protocol was developed by adapting existing literature procedures [24,28], similarly to what was done for WHC and WSC [24]. Briefly, 1 g of sample was mixed with 40 g of oil and left for 24 h at 25 °C. The mixture was then centrifugated (5810, Eppendorf, Eppendorf, Germany) at 4000 rpm (rcf 3220× *g*) for 20 min. The supernatant was eliminated, and the (wet) mass was quantified. The *OHC* value was calculated according to Equation (3).

(3) $$OHC=\frac{m_{w}-m_{d}}{m_{d}}$$

where $m_{w}$ and $m_{d}$ are the mass (g) of the wet and dry fiber, respectively. The OHC of fiber was determined for the following: rice oil (R), Miglyol^®^ 812 N (M), curcumin-loaded rice oil (R/C), and curcumin-loaded Miglyol^®^ 812 N (M/C). Functional properties were determined in duplicate, and the results were averaged.

### 4.3. Preparation of Curcumin/Oil Solution

The oil phases were prepared by adding to each oil the amount of curcumin corresponding to the maximum solubility (estimated according to literature data [21,52]), i.e., 2.67 mg/mL for rice oil (samples R/C) and 3.56 mg/mL for Miglyol^®^ 812 N (samples R/M). The procedure adopted for solution preparation was similar to that already used by other authors [37]. Curcumin was dissolved in the oil via mixing (ARE6, Velp Scientifica, Usmate Velate, Italy) at 60 °C for 10 min in a water-bath-controlled condition. The obtained solution was then sonicated (USC300, VWR International, Radnor, PA, USA) at 40 °C for 15 min to allow the complete dissolution of curcumin.

### 4.4. Preparation of Emulgels

The studied formulations were prepared by varying the fiber fraction content in the water phase (2% *w*/*w*, 2.5% *w*/*w*, and 3% *w*/*w*), the type of oil (rice oil and Miglyol^®^ 812 N), and curcumin loading. All samples had 40% *w*/*w* of oil phase, which is an average value between a low-fat food (10–25% *w*/*w*) and a full-fat food (60–70% *w*/*w*). Sample IDs and compositions are reported in Table 6.

**Figure 11:**
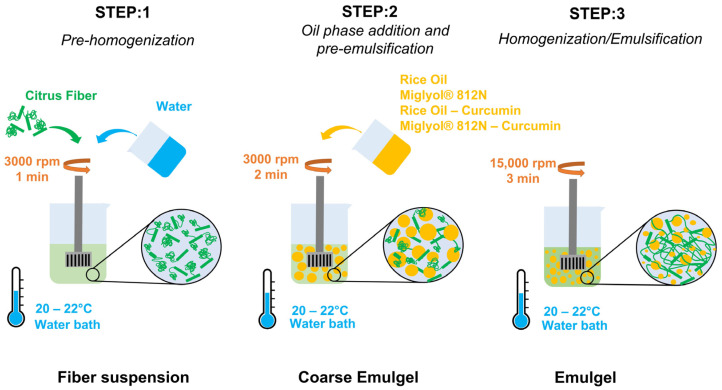
Schematic representation of emulgel preparation procedure.

Emulgel samples (batch of 50 g) were prepared though high-speed homogenization (HSH) using an Ultraturrax T25 (IKA-Werke GmbH & Co, Staufen, Germany) following a three-step protocol. First, citrus fiber powder was pre-homogenized in distilled water at 3000 rpm for 1 min, and subsequently, the oil phase was incorporated into the aqueous phase keeping the same rotational speed in approximately 2 min. After the addition of oil phases (either pure oil or curcumin oil solution), the speed was raised to 15,000 rpm and kept constant for 3 min. The sample temperature during processing was controlled by a water bath at 20–22 °C. The preparation procedure is schematically illustrated in Figure 11.

After the emulsification, samples were left in a fridge for 24 h at 5 °C and then characterized. For each concentration of fiber (2% *w*/*w*, 2.5% *w*/*w*, and 3% *w*/*w*), hydrogel samples were prepared as references following the same preparation steps (3 min 3000 rpm and 15,000 rpm for 3 min) and named H2, H2.5, and H3.

### 4.5. Rheological Characterization and Analysis

Rheological characterization was carried out with Haake MARS III (Thermo Fisher Scientific, Waltham, MA, USA) rheometer, equipped with a Peltier system for temperature control. The characterization of emulgel samples was carried out using serrated plate-plates geometry with a diameter of 35 mm (P35 TiL) and a measuring gap of 1.5 ± 0.1 mm. Frequency sweep tests at 25 °C were carried out between 0.1 and 10 Hz, in the linear region previously determined via a stress sweep test.

According to a rheological approach proposed in recent years [29,53,54], foods and weakly structured systems (such as bigels and emulgels) can be described as systems made by a three-dimensional network that behave as a critical gel in a limited frequency range, typically from 0.1 Hz up to 100 Hz; this is confirmed by the typical trend observed for dynamic moduli that are almost parallel with each other and linear in a log-log plot in the considered frequency range. According to this approach and to this model (named “weak gel model”) complex modulus, $G^{*}$ can be related to the frequency via a power law equation, Equation (4) [29].

(4) $$G^{*}=A\;\omega^{\frac{1}{z}}$$

where $G^{*}$ (Pa) is the complex modulus, ω (Hz) is the angular frequency, A (Pa s^z^) can be interpreted as a measure of the network strength, and z (dimensionless) is the network extension or coordination number and is related to the number of interacting rheological units forming the three-dimensional network. Flow curve tests were performed between 0.001 and 100 s^−1^ at 25 °C in duplicate, and the results were averaged. The viscosity data of flow curve tests were fitted using the modified form of the Cross model reported in Equation (5) [55].

(5) $$\eta =\frac{{µ}_{0}}{1+{\left(m\dot{\gamma }\right)}^{n}}$$

where η (Pa s) is the steady-state viscosity, $\dot{\gamma }$ (s^−1^) is the shear rate, ${µ}_{0}$ (Pa s) is the zero-shear viscosity, m (s) is the time constant, and n (dimensionless) is the shear-thinning index. With the purpose of investigating, in an exhaustive way, the rheology of samples and that of their components, frequency sweep tests (0.1–10 Hz) and a flow curve test (0.001–100 s^−1^) were performed at 25 °C on hydrogel samples (H2, H2.5, and H3), oils (R and M), and oil solutions (R/C and M/C). For hydrogels, the measuring geometry and gap were the same used for the emulgel, whereas cone-plate geometry with a diameter of 60 mm, an angle of 1°, and a gap at truncation of 0.052 mm (C50 TiL) was used for oils and oil solutions. Rheological measurements were performed twice, and the results were averaged.

### 4.6. Zeta Potential Measurements

The zeta potential (ζ) of citrus fiber and emulgel samples was evaluated using a Zetasizer Nano-ZS, Malvern Instruments Ltd. (Malvern, UK). To minimize multiple scattering effects, samples were properly diluted with distilled water immediately before analysis. The citrus fiber zeta potential was measured at 25 °C using a suspension of 10 mg of fiber in 100 mL. Emulgel samples (30 mg in 5 mL of distilled water) were analyzed at 25 °C, 37 °C, and 50 °C. The zeta potential (ζ) was determined for samples as prepared and after 24 h, 1, 2, and 3 weeks, and 1 month of storage at 5 °C. The ζ measurements were performed three times, and results are expressed as the mean value and standard deviation.

### 4.7. Optical Microscopy and Droplets Size Distribution (DSD) Analysis

Optical microscopy analyses, in contrast phase mode, were carried out with an MX5300H microscope, MEIJI (Saitama, Japan); micrographs were acquired at 20× magnification on emulgel samples to highlight the morphological features, whereas observations of diluted samples (1:20 *w*/*w*) were used to carry out a quantitative investigation of microstructure in terms of the droplet size distribution (DSD). In particular, the micrographs of diluted samples were used to determine the DSD by analyzing the images using Particle Analysis software dhs (version 1.1.0.9) (dhs image database, Greifenstein, Germany). The analysis consisted in the computation of the equivalent area circular diameter for each droplet in the micrographs. The parameters used for the quantitative description of DSD were 90th and 10th percentile diameters, $d_{90}$ and $d_{10}$, respectively; the median diameter, $d_{50}$; and the SPAN index, defined according to Equation (6).

(6) $$SPAN=\frac{d_{90}-d_{10}}{d_{50}}$$

For each diluted sample, 4 micrographs were acquired and processed to obtain the DSD, and parameters ($d_{10},\;d_{50},\;d_{90},\;SPAN$) were expressed as the mean value and standard deviation.

### 4.8. SEM and Cryo-SEM

The morphology of the dietary fiber powder sample was studied using a scanning electron microscope, FlexSem 1000 II (Hitachi, Tokyo, Japan). The micrographs of citrus fiber powder were acquired at 70× and 500× magnification using 5 kV as the accelerating voltage and 50 Pa as the (low) vacuum condition.

To assess the morphology of the water phase, as a reference, a micrograph of the (undiluted) hydrogel sample with 0.03 *w*/*w* of fiber (H3) was acquired at 500× using 10 kV as the accelerating voltage and 50 Pa as the pressure (low vacuum) and operating in cryo-mode (Coolstage, Deben, Bury St. Edmunds, UK). The morphology of selected emulgels was also investigated though electron microscopy in cryo-mode. Samples were diluted (1:10 *w*/*w*), and the micrographs were acquired in cryo-mode at 200× and 1000× with an accelerating voltage of 10–15 kV and using a pressure of 50 Pa (low vacuum). Hydrogel (H3) and emulgel samples were frozen at −35 °C using the cooling stage. This temperature was kept during all the scanning. Images were obtained from the signal associated with the backscattered electron (BSE), and no metallization of the surface of samples was applied for the observation.

### 4.9. Entrapment Efficiency and Loading Capacity

The entrapment efficiency (EE%, dimensionless) and loading capacity (LC, in mg/g) of emulgel samples were estimated using the method of Li and Wang [17] with a slight modification of the methanol-based demulsification procedure.

Briefly, 1 mL of methanol was mixed with 200 μL of a fresh prepared curcumin-loaded emulgel sample. The methanolic extract was then centrifuged (Fresco 17 Centrifuge, Thermo Fisher Scientific, Waltham, MA, USA) at 13,300 rpm (rcf 17,000× *g*) for 10 min at 25 °C. Next, the supernatant (methanolic solution), rich in curcumin, was recovered and further mixed with 1.8 mL of methanol before the UV/VIS analysis described later. Then, the entrapment efficiency (EE%) and loading capacity (LC) of the emulgel sample were determined according to Equation (7) and Equation (8), respectively

(7) $$EE\%=\frac{C_{tot}-C_{ext}}{C_{tot}}\times 100$$

where $C_{tot}$ (mg/mL) is the total amount of curcumin in the emulgel and C_ext_ (mg/mL) is the unloaded curcumin content in the emulgel, and

(8) $$LC=\frac{m_{c}}{m_{e}}$$

where $m_{c}$ (mg) is the mass of curcumin entrapped in the emulgel and $m_{e}$ (g) is the mass of the emulgel. Both EE% and LC were determined in triplicate and expressed as the mean value and standard deviation.

### 4.10. UV-VIS Spectroscopy

Spectrophotometric measurements in the UV-VIS range were carried out using an Evolution™ 201 UV-Visible Spectrophotometer (Thermo Fisher Scientific, Waltham, MA, USA) equipped with Thermo Scientific ^TM^ INSIGHT^TM^ Software (Version 2.3). Two calibration curves for curcumin were developed using methanol and a hydroethanolic solution (1:1 *v*/*v*) as solvents. For the former, the absorbance was measured at 424 nm [56] for the determination of the entrapment efficiency (EE%) and loading capacity (LC). For the hydroethanolic solution, the absorbance was recorded at 430 nm [57] to quantify curcumin release from the emulgel and oil solution samples. Stock solutions were prepared by dissolving 1 g of curcumin in 10 mL of methanol or a hydroethanolic solution (1:1 *v*/*v*) using a vortex (Zx3, Velp Scientifica, Usmate Velate, Italy) at 2000 rpm for 10 min and then sonicated in an ultrasonic bath (USC300, VWR International, Radnor, PA, USA) at 40 °C for 20 min [56]. Seven standard dilutions were then prepared from each stock solution to establish the respective calibration curves. The calibration curve of curcumin in methanol is reported in Figure S5a, whereas that of curcumin in hydroethanolic solution is presented in Figure S5b of the Supplementary Materials (SMs). For both calibration curves, a very good correlation coefficient (R^2^) was found (0.99321 and 0.99927, for methanol and hydroethanolic solution respectively).

### 4.11. In Vitro Release Analysis

In vitro drug release experiments were carried out over 24 h using Franz-type vertical glass diffusion (FD) cells having a receptor compartment of 8.5 mL and with an effective diffusion area (or cross-sectional surface area) of 0.567 cm^2^. The experiments were carried out using cellulose synthetic membranes (MWCO 12–14 kDa, Sigma-Aldrich, Saint Louis, MO, USA) mounted between the donor and receptor compartments. Samples (500 µL of curcumin-loaded emulgel or 200 µL of curcumin-loaded oil solution) were placed onto the membrane surface in the donor compartment and sealed with Parafilm^®^ to prevent evaporation. The receptor phase consisted of a hydroethanolic solution (50% *w*/*w* ethanol and 50% *w*/*w* ultrapure water) maintained at 37 °C by a circulating water jacket. The assembly was placed on a magnetic stirrer, and the receptor medium was continuously stirred using a magnetic bar. At scheduled time intervals, the receptor medium was completely withdrawn (8.5 mL) and replaced with an equal amount (ml) of fresh dissolution medium. Samples were analyzed using a spectrophotometer at 430 nm, and the cumulative percentage of curcumin released (Cur%) was calculated according to Equation (9).

(9) $$Cur\%=\left(\frac{M^{\prime }(t)}{M_{0}}\right)\times 100$$

where $M^{\prime }\left(t\right)$ is the cumulative released amount (in g), and it is defined as the sum of the quantified amounts of curcumin from *t*_0_ = 15 min to each time point (*t_i_*). $M_{0}$ it the total amount of curcumin in the sample (in g). Cur% at *t_i_* represents the cumulative release percentage at each sampling interval throughout the 24 h study. In vitro release tests were carried out in triplicate, and the results were averaged.

### 4.12. Statistical Analysis

All experimental data are shown in terms of the mean value and standard deviation.

Statistical analysis and data fitting were carried out using the software Origin Pro (Version 2021b; OriginLab Corporation, Northampton, MA, USA). A one-way ANOVA test was used to compare the values of the parameter of the weak gel model, modified Cross model, phase angle, and DSD parameters (90th and 10th percentile diameters, median diameter, and SPAN index); differences were assumed to be significant for *p* < 0.05, and a Tukey test was the method used for means comparison. A two-way analysis was used to compare the mean values of zeta potential for different storage times and temperatures; differences were assumed to be significant for *p* < 0.05, and a Tukey test was the method used for means comparison.

## Figures and Tables

**Table 1:** Physicochemical and functional properties of citrus fiber powder sample.

Property	Value
Humidity (%)	11.3 ± 0.3
Bulk density (g/mL)	0.456 ± 0.007
Particle size parameters	
10th percentile diameter, D10 (μm)	33
Median diameter, D50 (μm)	115
90th percentile diameter, D90 (μm)	287
ζ (mV)	−31 ± 1
Water Holding Capacity, WHC (g/g)	18.6 ± 0.9
Water Swelling Capacity, WSC (mL/g)	21 ± 1
Oil Holding Capacity, OHC (g/g)	
Rice oil	2.9 ± 0.1
Miglyol^®^ 812 N	2.85 ± 0.01
Rice oil + Curcumin (2.67 mg/mL)	2.86 ± 0.02
Miglyol^®^ 812 N + Curcumin (3.56 mg/mL)	2.85 ± 0.02

**Table 2:** Fitting parameters of weak gel model of emulgel samples at different fiber fractions. Different letters, for the same parameter, refer to significantly different values.

Emulgel ID	*A* (Pa s^z^)	*z* (−)	*δ* (°)
E2_R	260 ± 20 ^a^	16 ± 1 ^abc^	6.0 ± 0.1 ^a^
E2.5_R	444 ± 7 ^b^	13.53 ± 0.04 ^bc^	6.2 ± 0.1 ^a^
E3_R	750 ± 40 ^c^	18 ± 2 ^a^	5.83 ± 0.09 ^ab^
E2_M	199.1 ± 0.7 ^a^	16.9 ± 0.1 ^ab^	5.4 ± 0.1 ^b^
E2.5_M	371 ± 1 ^b^	15.0 ± 0.1 ^abc^	5.482 ± 0.002 ^b^
E3_M	597 ± 2 ^d^	14.8 ± 0.5 ^abc^	5.5 ± 0.1 ^b^

**Table 3:** Fitting parameters of modified Cross model of emulgels at different fiber fractions. Different letters, for the same parameter, refer to significantly different values.

Emulgel ID	µ_0_ (Pa s)	*m* (s)	*n* (−)
E2_R	2820 ± 60 ^a^	75 ± 2 ^a^	1.8 ± 0.1 ^ab^
E2.5_R	3600 ± 100 ^ab^	81 ± 4 ^a^	1.77 ± 0.02 ^ab^
E3_R	5000 ± 700 ^c^	82 ± 1 ^a^	1.85 ± 0.06 ^a^
E2_M	1130 ± 70 ^d^	124 ± 1 ^b^	1.73 ± 0.05 ^ab^
E2.5_M	2880 ± 32 ^a^	120 ± 7 ^b^	1.69 ± 0.08 ^ab^
E3_M	4600 ± 200 ^bc^	119 ± 8 ^b^	1.69 ± 0.07 ^ab^

**Table 4:** DSD parameters of emulgel samples. Different letters, for the same parameter, refer to significantly different values.

Emulgel ID	*d*_10_ (μm)	*d*_50_ (μm)	*d*_90_ (μm)	*SPAN*
E2_R	1.7 ± 0.5 ^a^	9 ± 3 ^ab^	19 ± 4 ^a^	2.3 ± 0.9 ^a^
E2.5_R	1.6 ± 0.3 ^a^	5 ± 2 ^ab^	21 ± 2 ^ab^	4 ± 1 ^abc^
E3_R	1.7 ± 0.6 ^a^	7 ± 2 ^ab^	21 ± 4 ^abc^	3 ± 1 ^ab^
E3_R/C	1.18 ± 0.05 ^a^	4.4 ± 0.9 ^b^	20 ± 3 ^a^	4.3 ± 0.7 ^abc^
E2_M	1.4 ± 0.2 ^a^	4.5 ± 0.4 ^b^	30 ± 10 ^abc^	7 ± 2 ^c^
E2.5_M	1.5 ± 0.4 ^a^	11 ± 6 ^a^	40 ± 10 ^c^	4 ± 2 ^abc^
E3_M	1.3 ± 0.2 ^a^	6 ± 1 ^ab^	34.8 ± 0.6 ^bc^	6 ± 1 ^bc^
E3_M/C	1.3 ± 0.1 ^a^	3.5 ± 0.5 ^b^	18 ± 6 ^a^	5 ± 2 ^abc^

**Table 5:** Kinetic models for curcumin release from oil solutions and emulgel samples. $M^{\prime }\left(t\right)$: cumulative released amount at time t; $M_{0}$ it the total amount of curcumin in sample; t: release time; $k_{0}$: zero order release rate constant; $k_{1}$: first order release rate constant; $k_{H}^{\prime }$: Higuchi release rate constant; $k_{KP}$: Korsmeyer–Peppas release rate constant; n: Korsmeyer–Peppas release exponent.

Kinetic Models	Equation		Samples		
		R/C	E3_R/C	M/C	E3_M/C
Zero-order	$\frac{M^{\prime }(t)}{M_{0}}=k_{0}t$	$k_{0}$ = (138 ± 2) × 10^−4^ h R^2^ = 0.99647 RSS = 0.00048 RMSE = 0.00635 AIC = −119 BIC = −119	$k_{0}$ = (76.2 ± 0.7) × 10^−4^ h R^2^ = 0.99899 RSS = 0.00004 RMSE = 0.00187 AIC = −127 BIC = −121	$k_{0}$ = (87 ± 3) × 10^−4^ h R^2^ = 0.98390 RSS = 0.00088 RMSE = 0.00855 AIC = −112 BIC = −112	$k_{0}$ = (59 ± 1) × 10^−4^ h R^2^ = 0.99377 RSS = 0.00016 RMSE = 0.00360 AIC = −133 BIC = −133
First-order	$\frac{M^{\prime }(t)}{M_{0}}=1-e^{-k_{1}t}$	$k_{1}$ = (160.1 ± 0.8) × 10^−4^ h R^2^ = 0.99967 RSS = 0.00003 RMSE = 0.00155 AIC = −153 BIC = −153	$k_{1}$ = (82 ± 1) × 10^−4^ h R^2^ = 0.99623 RSS = 0.00011 RMSE = 0.00298 AIC = −140 BIC = −140	$k_{1}$ = (95 ± 3) × 10^−4^ h R^2^ = 0.98582 RSS = 0.00044 RMSE = 0.00605 AIC = −121 BIC = −121	$k_{1}$ = (62 ± 2) × 10^−4^ h R^2^ = 0.98592 RSS = 0.00026 RMSE = 0.00462 AIC = −127 BIC = −127
Higuchi	$\frac{M^{\prime }(t)}{M_{0}}=k_{H}^{\prime }t^{1/2}$	$k_{H}^{\prime }$ = 0.047 ± 0.003 h^−1/2^ R^2^ = 0.79867 RSS = 0.01616 RMSE = 0.03670 AIC = −77 BIC = −77	$k_{H}^{\prime }$ = 0.02521 ± 0.003 h^−1/2^ R^2^ = 0.74586 RSS = 0.00679 RMSE = 0.02379 AIC = −88 BIC = −87	$k_{H}^{\prime }$ = 0.030 ± 0. 003 h^−1/2^ R^2^ = 0.85499 RSS = 0.00411 RMSE = 0.01851 AIC = −94 BIC = −93	$k_{H}^{\prime }$ = 0.019 ± 0.003 h^−1/2^ R^2^ = 0.69741 RSS = 0.00525 RMSE = 0.02092 AIC = −91 BIC = −90
Korsmeyer-Peppas	$\frac{M^{\prime }(t)}{M_{0}}={k_{KP}\;t}^{n}$	$k_{KP}$ = (172 ± 5) × 10^−4^ h^−0.92^ *n* = 0.92 ± 0.01 R^2^ = 0.99893 RSS = 0.00008 RMSE = 0.00254 AIC = −139 BIC = −138	$k_{KP}$ = (73 ± 3) × 10^−4^ h^−1.02^ *n* = 1.02 ± 0.01 R^2^ = 0.99848 RSS = 0.00004 RMSE = 0.00175 AIC = −148 BIC = −147	$k_{KP}$ = (141 ± 3) × 10^−4^ h^−0.824^ *n* = 0.825 ± 0.009 R^2^ = 0.99914 RSS = 0.00002 RMSE = 0.00135 AIC = −155 BIC = −154	$k_{KP}$ = (43 ± 2) × 10^−4^ h^−1.11^ *n* = 1.11 ± 0.02 R^2^ = 0.99841 RSS = 0.00002 RMSE = 0.00144 AIC = −153 BIC = −152

**Table 6:** Sample ID and composition.

Emulgel ID	% *w*/*w* of Citrus Fiber in Water Phase	Oil Type	Oil Phase ID	Curcumin (mg/mL)
E2_R	2	Rice Oil	R	-
E2_M	2	Miglyol^®^ 812 N	M	-
E2.5_R	2.5	Rice Oil	R	-
E2.5_M	2.5	Miglyol^®^ 812 N	M	-
E3_R	3	Rice Oil	R	-
E3_M	3	Miglyol^®^ 812 N	M	-
E3_R/C	3	Rice Oil	R/C	2.67
E3_M/C	3	Miglyol^®^ 812 N	M/C	3.56

## Data Availability

The data presented in this study are available upon request from the corresponding authors.

## Supplementary Materials

The following supporting information can be downloaded at: https://www.mdpi.com/article/10.3390/gels12050444/s1, Table S1. Typical zeta potential values of insoluble dietary fibers, Figure S1: Rheology of single phases: (a) frequency sweep test of hydrogels, (b) frequency sweep test of oils and oils phases, (c) flow curve test of hydrogels, and (d) flow curve test of oils and oil phase; Figure S2: Zeta potential (ζ) analysis of unloaded rice oil-based emulgels: (a) ζ of sample E2_R and (b) ζ of sample E2.5_R as a function of time and temperature; Figure S3: Zeta potential (ζ) analysis of unloaded Miglyol^®^ 812 N-based emulgels: (a) ζ of sample E2_M and (b) ζ of sample E2.5_M as a function of time and temperature; Figure S4: Cumulative release of curcumin from oil solutions and emulgel samples; Figure S5: Calibration curves for spectrophotometric analyses: (a) curcumin/methanol and (b) curcumin/hydroethanolic solutions. References [58,59,60] are cited in the supplementary materials.

## Abbreviations

The following abbreviations are used in this manuscript:

R	Rice oil
M	Miglyol^®^ 812N
R/C	Curcumin solution in rice oil
M/C	Curcumin solution in Miglyol^®^ 812N
LCT	Long Chain Triglycerides
MCT	Medium Chain Triglycerides
E2_R	Emulgel with rice oil and 2% *w*/*w* of fiber in water
E2_M	Emulgel with Miglyol^®^ 812N and 2% *w*/*w* of fiber in water
E2.5_R	Emulgel with rice oil and 2.5% *w*/*w* of fiber in water
E2.5_M	Emulgel with Miglyol^®^ 812N and 2.5% *w*/*w* of fiber in water
E3_R	Emulgel with rice oil and 3% *w*/*w* of fiber in water
E3_M	Emulgel with Miglyol^®^ 812N and 3% *w*/*w* of fiber in water
E3_R/C	Emulgel with rice oil and 3% *w*/*w* of fiber in water and curcumin
E3_M/C	Emulgel with Miglyol^®^ 812N and 3% *w*/*w* of fiber in water and curcumin
O/W	Oil in Water
W/O	Water in Oil
RSS	Residual Sum of Square
RMSE	Root Mean Squared Error
AIC	Akaike Information Criterion
BIC	Bayesian Information Criterion
WHC	Water Holding Capacity
WSC	Water Swelling Capacity
OHC	Oil Swelling Capacity
rpm	Revolution Per Minute
rcf	Relative Centrifugal force
EE%	Entrapment Efficiency
LC	Loading Capacity
MWCO	Molecular Weight Cut Off
Cur%	Percentage Cumulative Release of Curcumin
DSD	Droplets Size Distribution
SEM	Scanning Electron Microscopy
Cryo-SEM	Cryo-Scanning Electron Microscopy
UV-Vis	Ultra Violet-Visible
