# Supplementary material for: Structure–Function Relationship in Citrus-Fiber-Based Emulgels for Controlled Curcumin Delivery
Source: Gels. 2026 May 19;12(5):444. doi: 10.3390/gels12050444 (PMC13206092; doi:10.3390/gels12050444)
Supplement: Supplementary file 1 [file gels-12-00444-s001.zip › gels-4268535-supplementary.pdf]

Supplementary Materials (SM)

Structure–Function Relationship in Citrus Fiber-Based Emulgels for Controlled Curcumin Delivery

Domenico Mammolenti [1,*], Domenico Gabriele [1,*], Francesca Romana Lupi [1], Noemi Baldino [1] and Patrizia Formoso [2]

[1] Department of Information, Modeling, Electronics and Systems, (D.I.M.E.S.) University of Calabria, Via P. Bucci, Cubo 39C, 87036 Rende, Italy; francesca.lupi@unical.it (F.R.L); noemi.baldino@unical.it (N.B.)
[2] Department of Pharmacy, Health and Nutritional Sciences, University of Calabria, via A. Savino, Polyfunctional Building, 87036 Rende, Italy; patrizia.formoso@unical.it
* Correspondence: domenico.mammolenti@unical.it; Tel.: +39-0984-496675 (D.M.); domenico.gabriele@unical.it; Tel.: +39-0984-496687 (D.G.).

Insoluble dietary fibers	Type/Treatment	ζ (mV)	References
Citrus fiber	Native and conditioned (slat, sweeteners)	-6 to -38	[24]
Pomelo peel	Native and modified (acetylation, enzymatic hydrolysis, and ball milling)	-18 to -32	[58]
Pomelo peel	Native and modified (ball milling)	-20 to -35	[59]
Okara	Native and Modified (enzymatic/ultrasound)	-25 to -35	[60]

Table S1. Typical zeta potential values of insoluble dietary fibers.

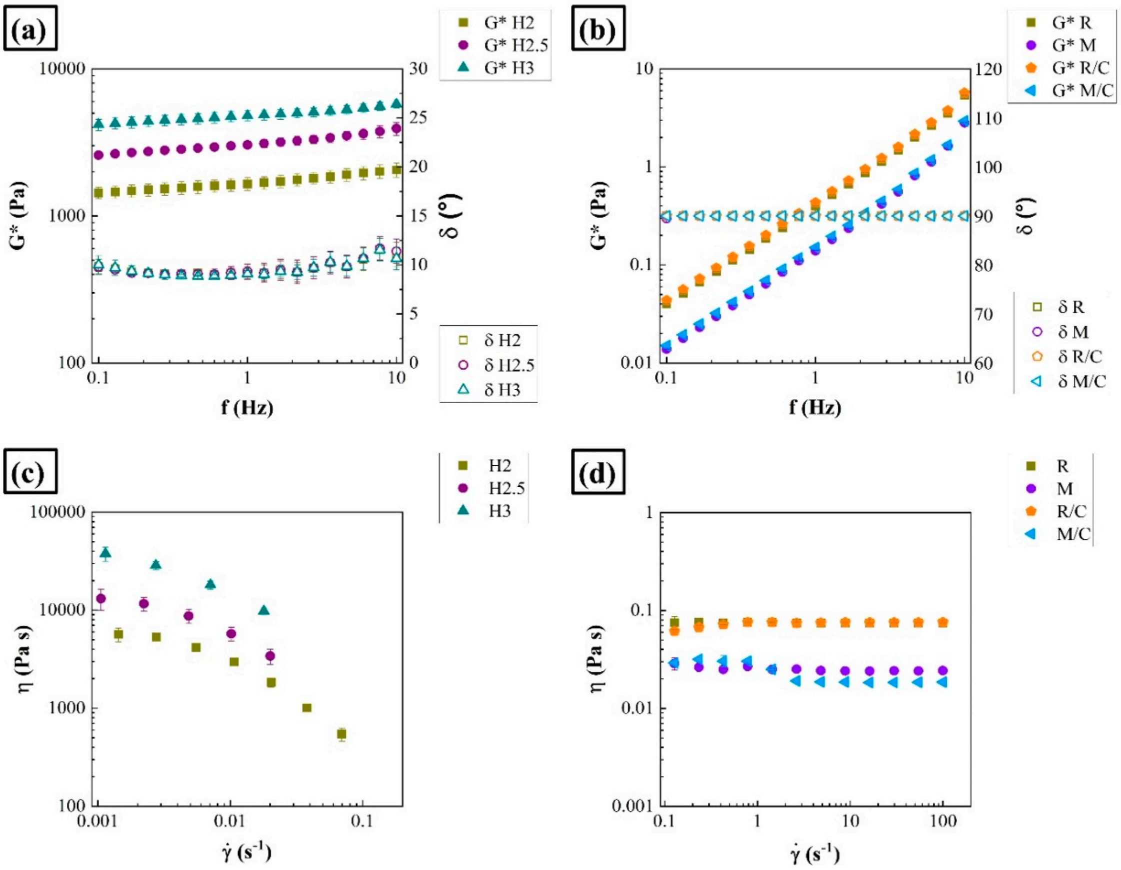

Figure S1. Rheology of single phases: (a) frequency sweep test of hydrogels, (b) frequency sweep test of oils and oils phases, (c) flow curve test of hydrogels and (d) flow curve test of oils and oil phases.

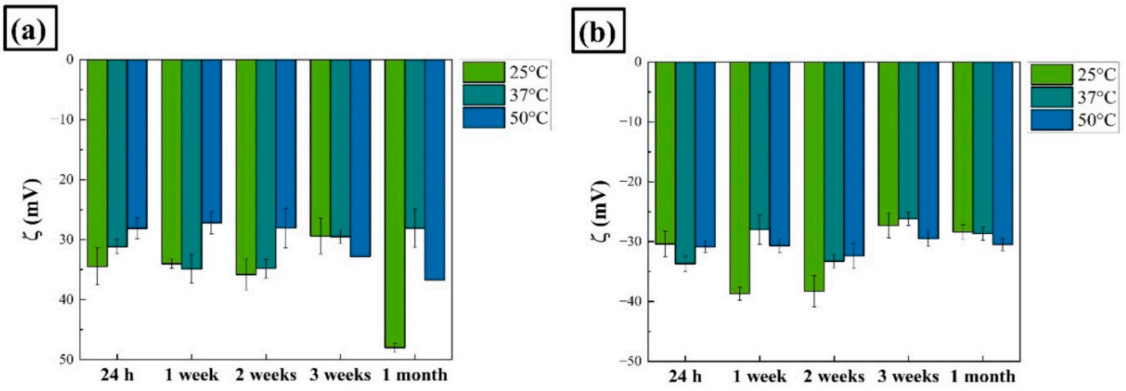

Figure S2. Zeta potential (ζ) analysis of unloaded rice oil-based emulgels: (a) ζ of sample E2_R and (b) ζ of sample E2.5_R as a function of time and temperature.

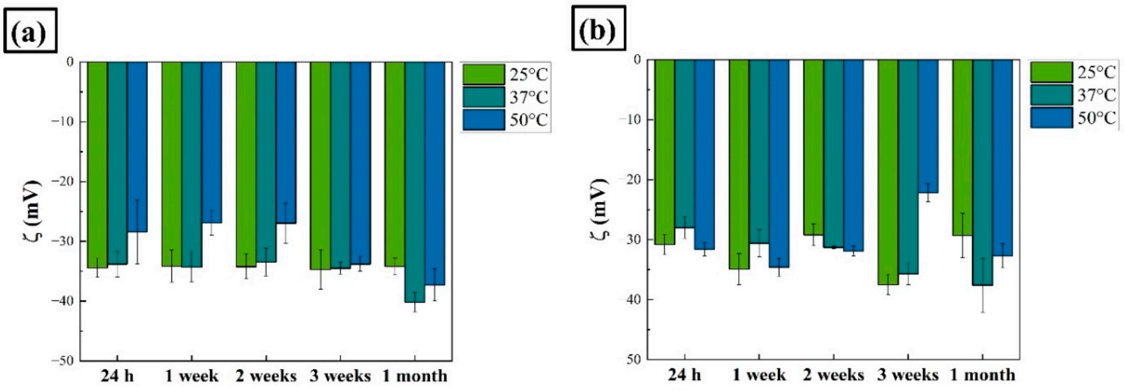

Figure S3. Zeta potential (ζ) analysis of unloaded Miglyol® 812N-based emulgels: (a) ζ of sample E2_M and (b) ζ of sample E2.5_M as a function of time and temperature.

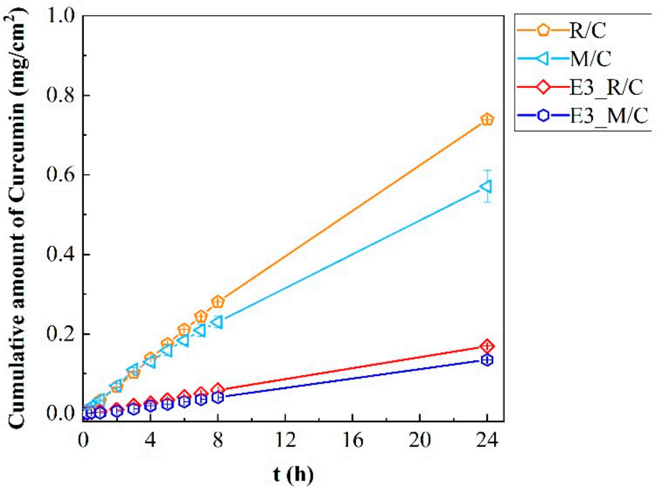

Figure S4. Cumulative release of curcumin from oil solutions and emulgel samples. Lines are intended as a visual aid.

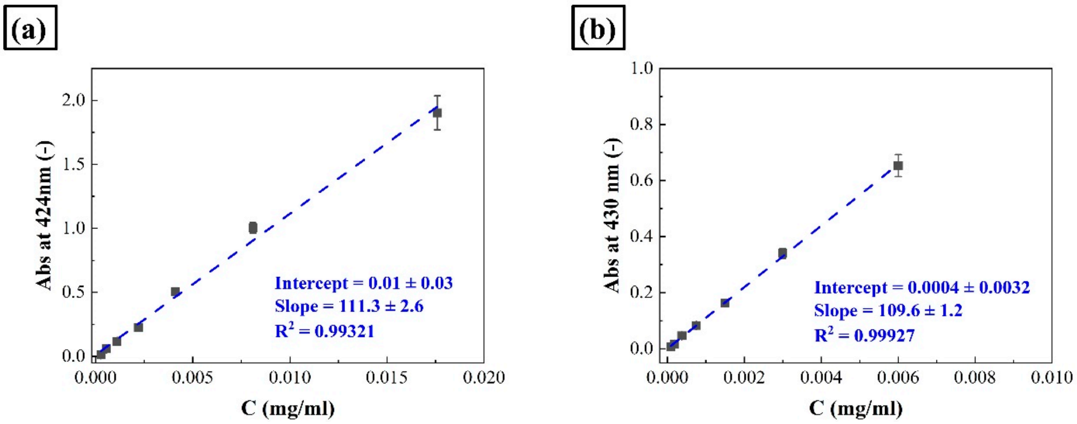

Figure S5. Calibration curves for spectrophotometric analyses: (a) curcumin/methanol and (b) curcumin/hydroethanolic solutions.

References

24. Mammolenti, D.; Lupi, F.R.; Bruno, E.; D'Agostino, A.; Mileti, O.; Baldino, N.; Gabriele, D. Impact of Solutes and Temperature on Rheological and Physical Properties of Particle Gels from Insoluble Dietary Fiber. Food Research International 2025, 221, https://doi:10.1016/j.foodres.2025.117256.
58. Yang, K.; Yao, J.; Shi, K.; Yang, C.; Xu, Y.; Zhang, P.; Pan, S. Emulsification Characteristics of Insoluble Dietary Fibers from Pomelo Peel: Effects of Acetylation, Enzymatic Hydrolysis, and Wet Ball Milling. Foods 2024, 13, 624. https://doi.org/10.3390/foods13040624.
59. Ke, J.;Wang, X.; Gao, X.; Zhou, Y.; Wei, D.; Ma, Y.; Li, C.; Liu, Y.; Chen, Z. Ball Milling Improves Physicochemical, Functionality, and Emulsification Characteristics of Insoluble Dietary Fiber from Polygonatum sibiricum. Foods 2024, 13, 2323. https://doi.org/10.3390/foods13152323.
60. Bao, Y.; Xue, H.; Yue, Y.; Wang, X.; Yu, H.; Piao, C. Preparation and Characterization of Pickering Emulsions with Modified Okara Insoluble Dietary Fiber. Foods 2021, 10, 2982. https://doi.org/10.3390/foods10122982.
